# Über ein Jahr B-Zell-gerichtete Therapie mit Ofatumumab s.c.: erste Ergebnisse einer prospektiven, patientenzentrierten Real-world-Beobachtungsstudie

**DOI:** 10.1007/s00115-023-01470-y

**Published:** 2023-04-12

**Authors:** Rafael Klimas, Anna-Sophia Karl, Philip Lennart Poser, Melissa Sgodzai, Simon Theile-Ochel, Barbara Gisevius, Simon Faissner, Ilias Nastos, Ralf Gold, Jeremias Motte

**Affiliations:** 1 Klinik für Neurologie, St. Josef-Hospital, Ruhr-Universität Bochum, Gudrunstraße 56, 44791 Bochum, Deutschland; 2 Facharztpraxis für Neurologie, Bochum, Deutschland

**Keywords:** Kesimpta, Multiple Sklerose, Immuntherapie, Anti-CD20-Therapie, Basistherapie, Kesimpta, Multiple sclerosis, Anti-CD20-Therapy, Patient reported outcomes, Immunotherapy

## Abstract

**Einleitung:**

Ofatumumab (Kesimpta™) ist ein s.c. anwendbarer Anti-CD20-Antikörper, welcher seit 2021 in Deutschland für die Behandlung der schubförmigen Multiplen Sklerose (RMS) eingesetzt wird. Die Selbstanwendung bietet ein hohes Maß an Unabhängigkeit von intravenösen Applikationsformen bei stark wirksamer Immuntherapie. In dieser Studie erfassten wir die patientenzentrierte Erfahrung bei 99 von 127 Patienten, die durch uns auf das Medikament eingestellt wurden. Ziel war die Untersuchung der Verträglichkeit und Akzeptanz aus Patientensicht.

**Methoden:**

Die Datensammlung erfolgte mittels Arztdokumentationen, Fragebögen und Telefoninterviews.

**Ergebnisse:**

Die Kohorte besteht aus 127 Patienten. Die Patienten erhielten 2,8 (± SD 1,7) Vortherapien. Die mittlere Therapiedauer mit Ofatumumab betrug 9,8 Monate (± SD 3,5). Strukturiert erfasst wurden 99 Patienten. 23 % der Patienten gaben an, während der Erstapplikation des Medikaments keine Nebenwirkungen gehabt zu haben. 19 % bewerteten die Nebenwirkungen als „sehr mild“ und 18 % als „mild“. Hierbei traten Schüttelfrost/Fieber (48 %), Kopf- (46 %) und Gliederschmerzen (45 %), sowie andere Symptome (19 %) auf. Bei Folgeinjektionen gaben 72 % der Patienten keine Nebenwirkungen an. 87 % der Patienten empfanden die Handhabung des Medikaments als „sehr einfach“. Es kam zu einem Schubereignis während der Therapie mit Ofatumumab.

**Diskussion:**

Unsere Studie zeigt, dass Ofatumumab von den Patienten gut akzeptiert und vertragen wird. Im Beobachtungszeitraum ist es zu einem Schubereignis gekommen. Die Nebenwirkungen sind mild und treten vor allem während der Erstanwendung auf. Es konnte keine erhöhte Infektneigung beobachtet werden. Die Daten legen nahe, dass Ofatumumab auch in der *Real-world*-Anwendung eine wirksame und sichere Therapieoption für Patienten mit einer schubförmig remittierenden multiplen Sklerose ist.

## Abstract (en)

**Introduction:**

Ofatumumab (Kesimpta™) is a s.c. applicable anti-CD20 antibody, which has been used in Germany since 2021 for the treatment of relapsing multiple sclerosis (RMS). The self-application offers a high degree of independence from intravenous forms of application with highly effective immunotherapy. In this study we recorded the patient-centered experience in 99 out of 127 patients who were adjusted to the drug by us. The aim was to investigate the tolerability and acceptance from the patient’s perspective.

**Methods:**

Data collection was carried out using doctor documentation, questionnaires and telephone interviews.

**Results:**

The cohort consists of 127 patients. The patients received 2.8 (± SD 1.7) pre-therapies. The mean duration of therapy with Ofatumumab was 9.8 months (± SD 3.5). Structured data were collected from 99 patients. 23% of patients had no side effects during initial application. 19% rated the side effects as “very mild” and 18% as “mild”. In addition to chills/fever (48%), headache (46%), limb pain (45%) and “other symptoms” (19%) also occurred. For subsequent injections, 72% of patients reported no side effects. 87% of patients found handling the medication “very easy”. There was one relapse event during therapy.

**Conclusion:**

Our study shows that Ofatumumab is well accepted and tolerated by patients. There was one relapse event during the observation period. The side effects are mild and occur during initial application. No increased tendency to infection could be observed. The data suggest that Ofatumumab is also an effective and safe treatment option for patients with relapsing remitting multiple sclerosis in real-world use.

## Einleitung

Subkutane Applikationsformen von hochaktiven Medikamenten zur Behandlung der multiplen Sklerose (MS) sind neue wichtige Bausteine einer modernen MS-Therapie. Sie ermöglichen eine niederschwellige und frühe Anwendung hocheffektiver Substanzen. Der frühe Einsatz hocheffektiver Substanzen hat zudem einen guten Effekt auf die Reduktion des Risikos von Progression [9, 13]. Der monoklonale Antikörper Natalizumab und B‑Zell-gerichtete Anti-CD20-Therapien waren bis 2021 nur in intravenöser Applikationsform verfügbar. Für Patienten war diese Form der Anwendung potenziell belastend, zeitaufwendig und mit hohem gesundheitsökonomischem Aufwand verbunden. Durch die subkutane Applikationsform sowohl von Natalizumab als auch der B‑Zell-gerichteten Therapie mit Ofatumumab können hocheffektive MS-Therapeutika weniger invasiv und teils durch die Patienten selbst appliziert werden.

Progression stellt die größte Herausforderung moderner MS-Therapien dar [7]. Eine zunehmende Behinderung kann auch aus einer schubunabhängigen Progression (PIRA) resultieren [14]. Der frühe Beginn einer hocheffektiven Therapie ist aktuell die einzige Möglichkeit, nachweislich Progression und somit Behinderung zu verhindern [3, 9, 13, 14].

Zu den Medikamenten der höchsten Wirksamkeitskategorie zählen neben Natalizumab und Alemtuzumab insbesondere auch die B‑Zell-adressierenden Therapien.

Mit Ocrelizumab wurde im Jahr 2019 erstmals ein B‑Zell-gerichteter humanisierter Antikörper für die MS-Therapie zugelassen. Mit Ofatumumab (Kesimpta™) steht seit September 2021 eine weitere B‑Zell-gerichtete Therapie für die schubförmige MS (RMS) in Deutschland zur Verfügung. Hierbei handelt es sich um einen voll humanen Anti-CD20-Antikörper. Die subkutane Selbstapplikation ermöglicht dem Patienten hierbei eine hohe Autonomie. In den Phase-III-Studien ASCLEPIOS I und II [8] zeigte sich eine signifikante Reduktion der Schubrate und der Behinderungsprogression im Vergleich zu Teriflunomid.

Die Zulassung von Ofatumumab erfolgte als „Arzneimittel unter zusätzlicher Überwachung – Schwarzes Dreieck“. Das heißt, Patientenakzeptanz, *Real-world*-Verträglichkeit sowie etwaige Nebenwirkungen unter Langzeitapplikation bedürfen noch weiterer Untersuchungen.

Die Ergebnisse unserer systematischen Erfassung demonstrieren aus patientenzentrierter Sicht die Verträglichkeit und Akzeptanz von Ofatumumab und sind wichtige zusätzliche Ergänzungen der Zulassungsstudien.

## Methodik

Unsere Beobachtungsstudie hatte das Ziel, die Verträglichkeit und Akzeptanz von Ofatumumab aus patientenzentrierter Sicht zu untersuchen.

Die Kohorte umfasst alle Patienten an der Universitätsklinik für Neurologie, Standort St. Josef Hospital Bochum, und Patienten einer sehr großen MS-Facharztpraxis im Ruhrgebiet (Dr. med. Nastos), die zwischen September 2021 und November 2022 auf eine MS-Therapie mit Ofatumumab eingestellt wurden, teilnehmen wollten und mindestens drei Monate mit Ofatumumab behandelt wurden. Die Einstellung auf Ofatumumab erfolgte gemäß der Fachinformation mit einer initialen Eindosierungsphase. Die Therapieentscheidung erfolgte durch den behandelnden Arzt bzw. die behandelnde Ärztin und wurde unabhängig von der Studie getroffen.

Die systematische Erfassung der Daten umfasste folgende Punkte:

- Soziodemografische Daten
- Krankheitsspezifische Daten
- Daten zur Therapie mit Ofatumumab
- Selbstauskunftsbögen (BDI, MSIS-29, SF-36, FSMC)

Darüber hinaus wurde der *Multiple-Sclerosis-functional-composite*-Test (MSFC) durchgeführt und die aktuelle *Expanded Disability Status Scale* (EDSS) ermittelt. Fehlende oder inkomplette Datensätze wurden mittels Telefoninterviews auf Basis der Fragebögen ergänzt. Gleichzeitig erfolgte eine systematische Analyse der klinikinternen Arztdokumentationen.

Bezüglich der Medikation mit Ofatumumab wurden insbesondere frühe Nebenwirkungen (innerhalb von 48 h post injectionem [p.i.]) sowie späte Nebenwirkungen (während einer Woche p.i.) nach Erstanwendung und in den Folgeanwendungen evaluiert. Weiter wurden die subjektive Handhabung sowie der Einfluss auf die Lebensqualität, Gehstrecke, Konzentration, Stimmung, Müdigkeit und den subjektiven MS-Krankheitsverlauf erfasst. Außerdem wurde die Therapieadhärenz untersucht. Für den Fall des Therapieabbruchs wurden die Gründe erfragt.

Die Auswertung der Daten erfolgte mittels Microsoft Excel Version 2211 Build 16.0.15831.20098.

## Ergebnisse

### Repräsentative MS-Kohorte

**Figure 1:**
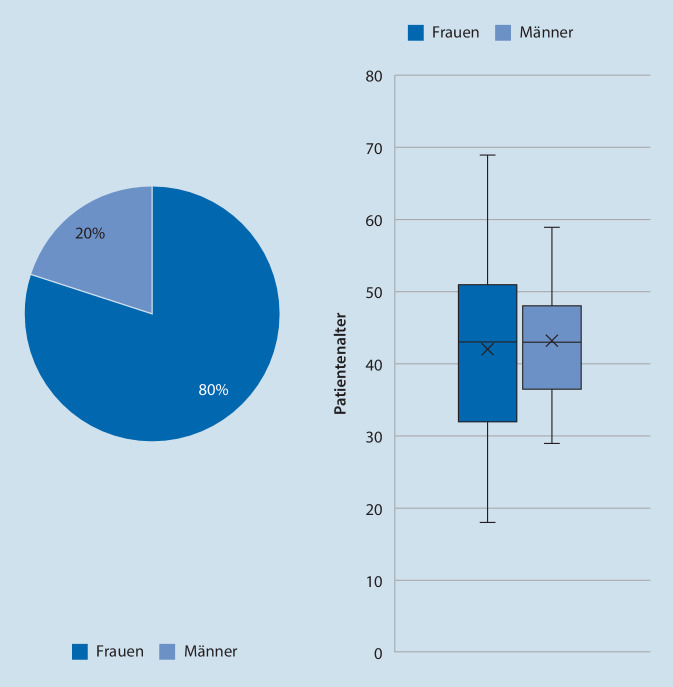

Im Zeitraum November 2021 bis November 2022 wurden insgesamt 127 Patienten neu auf Ofatumumab eingestellt. Hiervon konnten 78 % (*n* = 99) systematisch mittels Fragebogen, Telefoninterview und klinikinterner Arztdokumentation vollständig erfasst werden. Soziodemografisch zeigte sich eine Geschlechterverteilung von 80 % (*n* = 79) Frauen zu 20 % (*n* = 20) Männern. Das durchschnittliche Patientenalter betrug 42,2 Jahre (± Standardabweichung [SD] 11,2 Jahre, Abb. 1). Die durchschnittliche Erkrankungsdauer lag bei 9,1 Jahren (± SD 7,7). Bildungsstand, Berufssituation sowie die MS-spezifische Familienanamnese lassen sich aus Tab. 1 entnehmen.

**Table 1:** 

Domänen des Short Form 36	N	Minimum	Maximum	Mittelwert	SD
Körperliche Summenskala	68	14,18	58,70	41,77	10,74
Psychische Summenskala	68	22,81	62,86	45,46	10,29
Körperliche Funktionsfähigkeit	70	5,00	100,00	68,31	27,59
Körperliche Rollenfunktion	70	0,00	100,00	58,21	40,98
Körperliche Schmerzen	70	0,00	100,00	62,81	28,69
Allgemeine Gesundheitswahrnehmung	70	5,00	100,00	49,66	21,40
Vitalität	70	0,00	85,00	40,97	23,88
Soziale Funktionsfähigkeit	70	0,00	100,00	71,43	25,28
Emotionale Rollenfunktion	68	0,00	100,00	70,59	38,43
Psychisches Wohlbefinden	69	16,00	100,00	65,13	19,18

### Vortherapien und EDSS

**Figure 2:**
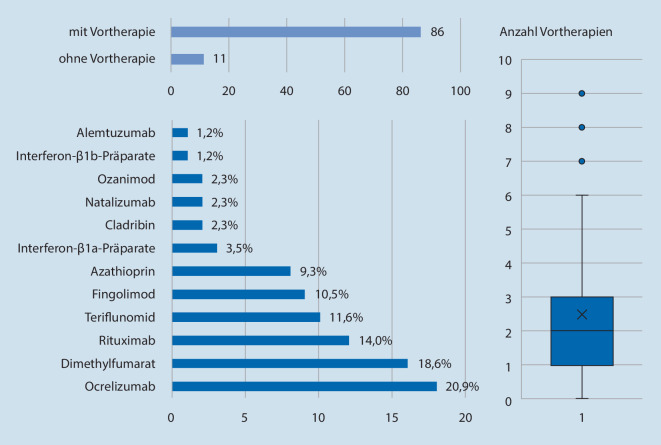

Im Durchschnitt haben die Patienten 2,8 (± SD 1,7) Vortherapien erhalten. Dabei war Ocrelizumab mit 18,6 % (*n* = 18) die häufigste Vortherapie. 11,3 % (*n* = 11) der Patienten hatten keine Vortherapie (Abb. 2). Der durchschnittliche EDSS-Wert lag bei 2,3 (± SD 1,1; Min. 1,0; Max. 6,5).

### Ein Schubereignis, zwei Therapieabbrüche

Während der Therapie mit Ofatumumab bestand bei einer Patientin ein Schubereignis nach Beginn der Therapie, sodass die Therapie bereits nach 4 Wochen umgestellt wurde. Ein weiterer Patient brach die Therapie aufgrund von Nebenwirkungen ab, sodass es insgesamt zu 2 (2,4 %) Therapieabbrüchen kam.

### Ofatumumab: hohe, aber überwiegend milde Nebenwirkungsrate bei Erstanwendung

**Figure 3:**
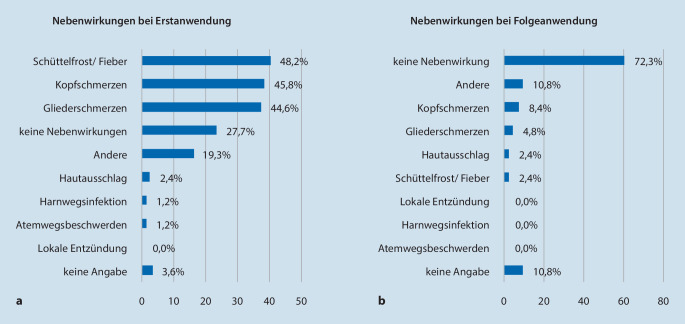

**Figure 4:**
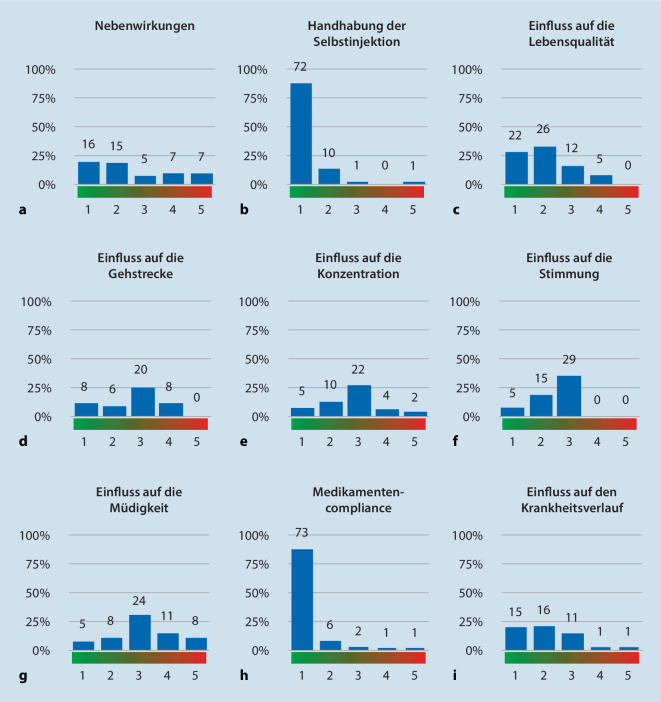

Die durchschnittliche Therapiedauer mit Ofatumumab betrug 9,8 Monate (± SD 3,5; Min. 3; Max. 15). Ausgehend von 83 vollständigen Datensätzen hatten 28 % (*n* = 23) der Patienten während der Erstapplikation von Ofatumumab keine Nebenwirkungen (Abb. 3a). 3 Patienten trafen diesbezüglich keine Aussage. 57 Patienten (69 %) berichteten in absteigender Häufigkeit von Schüttelfrost und Fieber (*n* = 40; 48 %), Kopfschmerzen (*n* = 38; 46 %), Gliederschmerzen (*n* = 37; 45 %), anderen Symptomen (*n* = 16; 19 %), Hautausschlag (*n* = 3; 4 %), Atemwegsbeschwerden (*n* = 1; 1 %) und Harnwegsinfektionen (*n* = 1; 1 %). Unter den „anderen“ Symptomen wurde am häufigsten Müdigkeit genannt. Im Vergleich dazu schilderten nur 14 Patienten (17 %) Nebenwirkungen während der Folgeanwendungen (Abb. 3b). 72 % (*n* = 60) gaben an, keine Nebenwirkungen mehr im Verlauf gehabt zu haben. 11 % (*n* = 9) trafen diesbezüglich keine Aussage. Während der Folgeanwendungen traten am häufigsten Symptome wie Müdigkeit auf (*n* = 9; 11 %). Genannt wurden zusätzlich Kopfschmerzen (*n* = 7; 8 %), Gliederschmerzen (*n* = 4; 5 %), Hautausschlag (*n* = 2; 2 %) sowie Schüttelfrost und Fieber (*n* = 2; 2 %). Die Schwere der Nebenwirkungen wurde von 19 % (*n* = 16) als „sehr mild“, 18 % (*n* = 15) als „mild“, 6 % (*n* = 5) als „neutral“, 8 % (*n* = 7) als „stark“ und 8 % (*n* = 7) als „sehr stark“ empfunden (Abb. 4a). 41 % (*n* = 34) der Patienten äußerten sich nicht zur Schwere der Nebenwirkungen.

### Ofatumumab: hohe Akzeptanz und positiver Eindruck aus Patientensicht

87 % (*n* = 72) der Patienten fiel die Handhabung des Medikaments „sehr einfach“, 10 (12 %) „einfach“. Je ein Patient empfand die Behandlung als „neutral“ (1 %) bzw. „sehr schwer“ (1 %; Abb. 4b).

Den Einfluss auf die Lebensqualität beschrieben 27 % (*n* = 22) als „sehr positiv“, 31 % (*n* = 26) als „positiv“. 15 % (*n* = 12) sahen weder einen positiven noch einen negativen Einfluss auf die Lebensqualität und beschrieben den Einfluss als „neutral“. 5 Patienten (6 %) empfanden einen negativen Einfluss auf die Lebensqualität, 17 (21 %) konnten diesbezüglich keine Meinung bilden und antworteten mit „ich weiß nicht“ (Abb. 4c).

Der Einfluss auf die Gehstrecke wurde von 39 Patienten (47 %) als unbekannt bewertet, 20 (24 %) bewerteten den Einfluss als „neutral“. 8 Patienten (10 %) gaben einen „sehr positiven“, 6 (7 %) einen „positiven“ Einfluss auf die Gehstrecke an. 10 % der Patienten (*n* = 8) gaben einen „negativen“ Einfluss auf die Gehstrecke an (Abb. 4d).

Bezüglich des Einflusses von Ofatumumab auf die Konzentrationsfähigkeit wussten 46 % der Patienten (*n* = 38) nicht, wie sie antworten sollten. 6 % (*n* = 5) beschrieben einen „sehr positiven“, 12 % (*n* = 10) einen „positiven“ Einfluss, 27 % (*n* = 22) empfanden ihre Konzentration „neutral“. 5 % (*n* = 4) gaben einen „negativen“ und 2 % (*n* = 2) einen „sehr negativen“ Einfluss auf die Konzentration an (Abb. 4e).

33 Patienten (40 %) bewerteten nicht, wie Ofatumumab auf ihre Stimmung wirkt. 35 % (*n* = 29) empfanden ihre Stimmung „neutral“, 6 % (*n* = 5) „sehr positiv“ und 18 % (*n* = 29) „positiv“. Eine „negative“ oder „sehr negative“ Bewertung der Stimmung wurde nicht genannt (Abb. 4f).

Die Müdigkeit unter Therapie mit Ofatumumab wurde von 24 (29 %) als „neutral“ bewertet. 13 % (*n* = 11) gaben „mehr Müdigkeit“ und 10 % (*n* = 8) „viel mehr Müdigkeit“ an. „Viel weniger Müdigkeit“ wurde von 5 Patienten (6 %) und „weniger Müdigkeit“ von 8 (10 %) genannt. 30 % (*n* = 25) konnten keine Aussage treffen (Abb. 4g).

Bezüglich der Medikamentencompliance gaben 88 % der Patienten (*n* = 73) an, sich „sehr stark“ an die monatlichen Selbstinjektionen zu halten. 7 % (*n* = 6) hielten sich „stark“, 2 % (*n* = 2) weder stark noch schwach, 1 Patient (1 %) hielt sich wenig an die Injektionsintervalle und ein weiterer „sehr wenig“ (1 %) daran (Abb. 4h).

Insgesamt bewerteten 15 Patienten (18 %) den Einfluss von Ofatumumab auf ihren MS-Krankheitsverlauf als „sehr positiv“, 16 (19 %) als „positiv“ und 11 (13 %) als „neutral“. Ein Patient (1 %) empfand den Einfluss des Medikaments auf den MS-Verlauf als „negativ“, ein weiterer Patient (1 %) als „sehr negativ“. 46 % (*n* = 38) konnten keine Angabe diesbezüglich treffen (Abb. 4i).

### „Patient-reported outcomes“ nach mind. 3 Therapiemonaten

#### MS-Beeinträchtigungsskala (MSIS-29)

**Figure 5:**
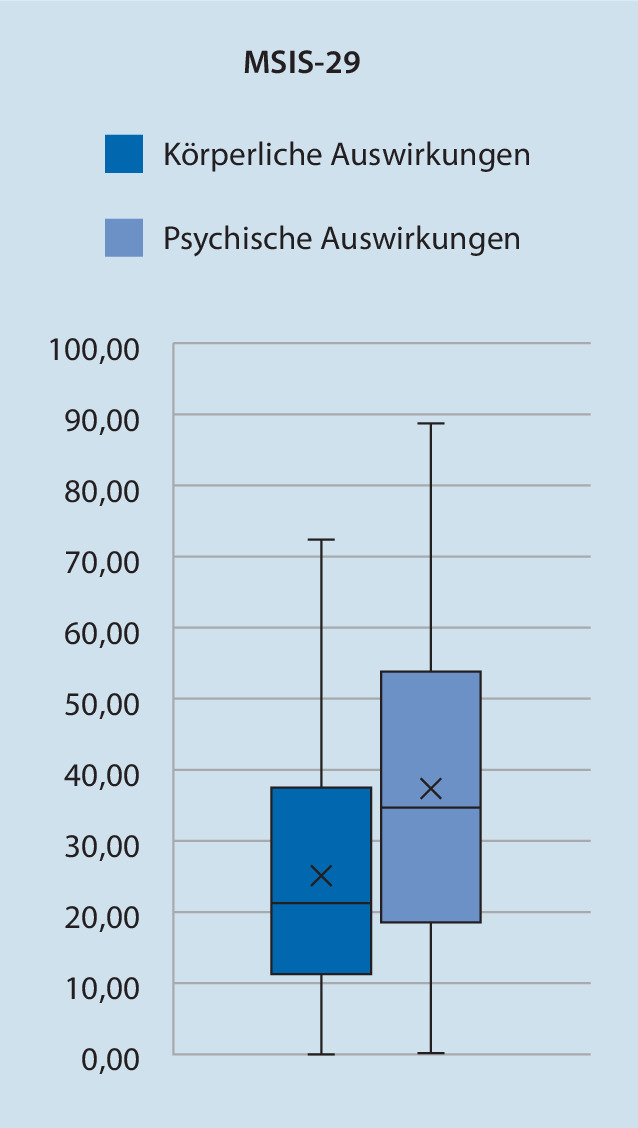

Mit MSIS-29 wurde der Einfluss der MS auf physische (*„physical impact score“*) sowie psychische (*„psychological impact score“*) Faktoren ermittelt und auf einer Skala von 0 (= kein Einfluss der Erkrankung) bis 100 (= maximaler Einfluss der Erkrankung) interpoliert. Ausgehend von *n* = 70 vollständigen Datensätzen erreicht die Kohorte im physischen Score einen Mittelwert ± SD von 25,1 ± 18,7 mit einem minimalen Score von 0 und einem maximalen Score von 72,5. Im psychologischen Score wird ein Mittelwert ± SD von 37,1 ± 23,0 mit einem minimalen Score von 0 und einem maximalen Score von 88,9 erreicht (siehe Abb. 5).

#### Fatigueskala für Motorik und Kognition (FSMC)

Die Schwere der Fatiguesymptomatik wurde mittels FSMC ermittelt. Hierbei wurde zwischen motorischer, kognitiver Fatigue und Gesamtfatigue unterschieden.

**Figure 6:**
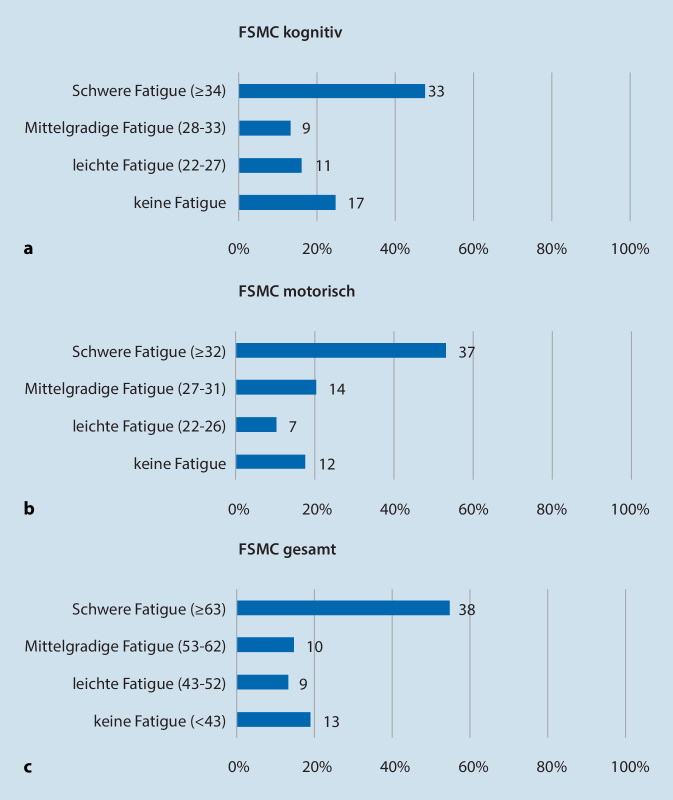

47 % der Patienten (*n* = 33) litten unter einer schweren kognitiven Fatigue, 13 % (*n* = 9) unter einer mittelschweren, 16 % (*n* = 11) unter einer leichten und 24 % (*n* = 17) litten unter keiner kognitiven Fatigue. Im Mittel erreichte die Kohorte eine Punktzahl von 30,2 ± 10,8 SD (Abb. 6a).

Unter einer schweren motorischen Fatigue litten 53 % der Patienten (*n* = 37), mittelschwer waren 20 % (*n* = 14) betroffen, leichtgradig 10 % (*n* = 7) und 17 % (*n* = 12) hatten keine motorische Fatigue. Im Mittel erreichte die Kohorte eine Punktzahl von 31,4 ± 9,9 SD, was einer mittelgradigen bis schweren motorischen Fatigue entspricht (Abb. 6b).

In der Gesamt-FSMC zeigten 38 Patienten (54 %) eine schwere Fatigue, 10 Patienten (14 %) eine mittelschwere, 9 (13 %) eine leichtgradige und 13 Patienten (19 %) keine Fatigue. Im Mittel erreichte die Kohorte eine Gesamtpunktzahl von 61,5 ± 20,0 SD, was einer mittelgradigen bis schweren Gesamtfatigue entspricht (Abb. 6c).

#### Beck Depression Inventar (BDI)

Die Häufigkeit einer Depression in unserer Studienkohorte wurde mittels BDI ermittelt. 40 % der Patienten (*n* = 28) hatten keine Depression, 14 % (*n* = 10) litten unter einer minimalen Depression, 23 % (*n* = 16) unter einer leichten Depression. Eine mittelschwere Depression trat bei 19 % (*n* = 13) auf, eine schwere bei 4 % (*n* = 3). Die Gesamtkohorte erreichte im Mittel ± SD 13,3 ± 9,6 Punkte, was einer minimalen bis leichtgradigen Depression entspricht.

#### Short Form 36 (SF-36)

Die Bewertung der Lebensqualität erfolgt über 8 Domänen der SF-36 und wird in einer körperlichen sowie psychischen Summenskala zusammengefasst.

Die Skalen bewegen sich zwischen 0 (= sehr geringe Lebensqualität) und 100 (= sehr hohe Lebensqualität) im Vergleich zu einer bundesdeutschen Normpopulation [5]. Insgesamt zeigen unsere Daten, dass unsere Kohorte sowohl in der körperlichen Summenskala (Mittelwert 41,8 ± 10,7 SD; Min. 14,2; Max. 58,7) als auch in der psychischen Summenskala (Mittelwert 45,5 ± 10,3 SD; Min. 22,8; Max. 62,9) eine krankheitsbezogen geringere Lebensqualität aufweist als eine bundesdeutsche Normpopulation (Tab. 2).

**Table 2:** 

Höchster Bildungsabschluss	n	%	Berufssituation	n	%	MS-Familienanamnese	n	%
Kein Abschluss	0	0,0	Aus‑/Weiterbildung	2	2,9	Großeltern	1	1,4
Hauptschule	5	7,1	Vollzeit	28	40,0	Eltern	2	2,9
Realschule	6	8,6	Teilzeit	20	28,6	Geschwister	0	0,0
Fachhochschule	7	10,0	Hausfrau/-mann	4	5,7	Eigene Kinder	0	0,0
Berufsausbildung	16	22,9	Berufs-/erwerbsunfähig	5	7,1	Enkelkinder	0	0,0
Bachelor	6	8,6	Altersrente	1	1,4	Keine	62	88,6
Master	8	11,4	Arbeitssuchend	2	2,9	Keine Angabe	5	7,1
Abitur	9	12,9	Keine Angabe	8	11,4	–	–	–
Staatsexamen	5	7,1	–	–	–	–	–	–
Keine Angabe	8	11,4	–	–	–	–	–	–

## Diskussion

Unsere Studie zeigt eine hohe Verträglichkeit und Akzeptanz von Ofatumumab in der subkutanen Anwendung. Damit können wir erstmalig mit *Real-world*-Patientendaten die zuvor berichtete hohe Verträglichkeit von Ofatumumab aus den randomisierten Zulassungsstudien bestätigen [1, 8, 18].

Ofatumumab zeigt mit der ersten Injektion eine hohe Nebenwirkungsrate. Die Nebenwirkungen sind jedoch mit einer milden Symptomatik verbunden und treten im Verlauf der Behandlung bei weniger als 20 % der Patienten auf. Die häufigsten Symptome sind Schüttelfrost und Fieber infolge der schnellen B‑Zell-Elimination [6]. Durch rasche Zytokinfreisetzung können die meisten Symptome erklärt werden. Die Milde dieser Nebenwirkung wird durch eine geringe Abbruchquote objektiviert – wir empfahlen ggf. die Einnahme von nichtsteroidalen Antiphlogistika, Flüssigkeitszufuhr und Ruhe. Gleichzeitig berichtet unsere Kohorte von einer nahezu nebenwirkungsfreien Folgeanwendung des Medikaments. Compliance und Akzeptanz sind sehr hoch, was möglicherweise an der einfachen Anwendung des Medikaments liegt (Abb. 4a). Eine erhöhte Infektneigung kann nicht beobachtet werden. Ebenso kam es im Beobachtungszeitraum nur bei einer Patientin zu einem Schubereignis. Diese Patientin erhielt zu diesem Zeitpunkt erst vier Wochen Ofatumumab. Die Therapieeinstellung erfolgte aufgrund eines sensiblen Schubereignisses. Nach vier Wochen erlitt die Patientin ein erneutes Schubereignis mit neu aufgetretenen supratentoriellen sowie spinalen Läsionen. Zuvor war die Patientin drei Monate ohne Basistherapie. Damit ist das Schubereignis aufgrund noch fehlender Therapiewirkung von Ofatumumab zu werten. Die volle immunologische Wirkung von Ofatumumab ist erst nach sechs bis acht Wochen zu erwarten.

Mit durchschnittlich ca. 3 Vortherapien und einem EDSS-Wert von 2,3 Punkten ist unsere Kohorte vordergründig mäßig krankheitsbelastet. Die Ergebnisse unserer *„patient-reported outcomes“* zeigen, dass die Kohorte trotz der relativ geringen Behinderung stark in verschiedenen Domänen beeinträchtigt ist. Wir können mittels SF-36 objektiv eine verminderte Lebensqualität insbesondere in der Domäne der Gesundheitswahrnehmung nachweisen. Dennoch berichtet ein Teil der Patienten subjektiv von einer Verbesserung der Lebensqualität unter Ofatumumab. Ob dieser subjektive Eindruck langfristig anhält, werden wir zukünftig mittels longitudinaler Daten der noch laufenden Beobachtung beurteilen.

Der langfristige Effekt der Therapie mit Ofatumumab, insbesondere auf Fatigue, Konzentration und Gehstrecke, ist zum aktuellen Zeitpunkt noch nicht beurteilbar. Jedoch berichtet ungefähr die Hälfte der Patienten auch in dieser frühen Phase erste subjektive Einschätzungen:

Hinsichtlich der Gehstrecke berichten die wenigsten Patienten von einer Verschlechterung unter Ofatumumab, während etwa 20 % einen positiven Effekt bemerken. Wie bei dem kurzen Beobachtungs- und Therapiezeitraum zu erwarten ist, berichten die meisten Patienten von keinem Einfluss auf die Gehstrecke. Da der durchschnittliche EDSS-Wert bei 2,3 liegt, ist unsere Kohorte insgesamt gering gehbeeinträchtigt. Trotzdem zeigt der physische Teil der MSIS-29 eine deutliche motorische Krankheitslast (Abb. 5). Dies kann möglicherweise durch die hohe motorische Fatigue erklärt werden; ein Befund, der bei früh erkrankten MS-Patienten bekannt ist [15]. Eine positive Bewertung der Gehstrecke kann infolgedessen auch ein Hinweis auf eine Verbesserung der Fatigue durch Ofatumumab sein, welche von gut 15 % unserer Patienten berichtet wird.

Subjektiv berichten die wenigsten Patienten, dass Ofatumumab negativ auf ihre Konzentration wirkt. Knapp 20 % schildern sogar eine Verbesserung ihrer Konzentrationsfähigkeit. Gleichzeitig zeigt die FSMC jedoch, dass etwa die Hälfte der Kohorte unter einer schweren kognitiven Fatigue leidet. Dies spiegelt sich auch in der Befragung hinsichtlich des Einflusses von Ofatumumab auf die Müdigkeit wider. Etwa ein Viertel der Patienten berichtet von „viel mehr“ bzw. „mehr“ Müdigkeit im Alltag. Inwiefern diese Angaben objektivierbar sind und ob Ofatumumab wirklich die kognitive Fatigue fördert, werden wir in weiteren longitudinalen Studien untersuchen.

Im Vergleich zwischen BDI und der berichteten Stimmungsänderung unter Ofatumumab zeichnet sich ein positiver Effekt ab. Etwa ein Viertel der Patienten berichtet von einer Stimmungsbesserung trotz hoher Depressionsrate im BDI. Insgesamt zeigt MSIS-29 im psychologischen Teil eine höhere psychische als körperliche Beeinträchtigung. Dies lässt sich vor allem durch die hohe kognitive Fatigue und die hohe Depressionsrate erklären [11, 12].

Der subjektive Therapieerfolg auf die Stimmung und Müdigkeit wird derzeit von den Patienten noch neutral beschrieben. Der Großteil kann keine exakte Einordnung zu diesen Themen treffen. Positive oder negative Effekte von Ofatumumab auf Fatigue und Depression könnten durch weitere Beobachtungszeitpunkte objektiviert werden. Dies gilt auch für den Einfluss des Medikaments auf den gesamten Krankheitsverlauf. Auch hier berichtet die Hälfte der Patienten, keine Antwort zu wissen. Positive Tendenzen zeichnen sich jedoch ab, da derzeit mehr als ein Drittel der Patienten eine subjektive positive Wirkung verspürt.

Die meisten Patienten unserer Kohorte empfinden positive Effekte durch Ofatumumab. Die positive Wahrnehmung kann auch allein durch die Applikationsform und durch das Wissen, dass eine hochaktive Substanz erhalten wird, begründet sein. Jedoch erhielt etwa ein Viertel der Patienten bereits eine hochaktive Vortherapie (Abb. 2). Für die wenigsten Patienten ist Ofatumumab die Ersttherapie und die meisten hatten schon vor Therapieumstellung einen stabilen MS-Verlauf. Aus diesen Gründen gehen wir davon aus, dass die positive Wahrnehmung von Ofatumumab keine primär immunologische Ursache hat. Die positive Wahrnehmung kann durch die einfache Handhabung und das Gefühl der Selbstbestimmung bzw. Selbstwirksamkeit der Patienten beeinflusst sein.

Das ideale MS-Medikament ist immunologisch effektiv, verhindert Progression, ist gut verträglich und hat keine gefährlichen Nebenwirkungen.

Vor 20 Jahren standen mit den *„injectables“*, Interferon-β-Analoga und Glatirameracetat erstmals spezifische MS-Medikamente zur Verfügung, welche bis heute als MS-Immuntherapeutika der geringsten Wirksamkeit eingesetzt werden. Mit ihrer schwachen immunologischen Wirkung im Vergleich zu hocheffektiven MS-Therapien und relativ schlechter Verträglichkeit ist ihr breiter Einsatz jedoch zunehmend umstritten [10, 19].

Hocheffektive Therapien sind mittlerweile gut verträglich und zeigen eine hohe immunologische Wirksamkeit. Ihr früher Einsatz verhindert nachweißlich Behinderungsprogression [2, 9, 18]. Der nationale Vergleich der beiden Therapiestrategien – früher Beginn hochaktiver Therapie (Schweden) gegen Eskalationsstrategie (Dänemark) – zeigt, dass der Beginn mit einer höher wirksamen Therapie eine Progression besser verzögert als der Beginn mit einer weniger effektiven Therapie [18]. Die Erstbehandlung mit hocheffektiven Therapien geht mit einem geringeren Risiko der Umwandlung in eine sekundär progrediente MS [2] und einer geringeren Behinderung in einem Zeitintervall von sechs Jahren einher [9]. Ihr potenziell höheres Nebenwirkungspotenzial ist durch inzwischen langjährige Erfahrung und gut etablierte Sicherheitsuntersuchungen kontrollierbar. Auch schwere Nebenwirkungen wie die progressive multifokale Leukenzephalopathie (PML) bei Natalizumab sind immer besser beherrschbar geworden [4, 17].

Damit ist in der praktischen Anwendung hochaktiver Therapien vor allem der hohe logistische Aufwand der Applikation ein Nachteil. Bis vor zwei Jahren waren sowohl Natalizumab als auch die B‑Zell-depletierende Therapie nur als intravenöse Applikation verfügbar.

Die subkutanen Applikationsformen verbessern ihre Anwendung aus patientenzentrierter Sicht und erleichtern damit ihre frühe Anwendung [16].

Diese patientenzentrierte Verbesserung kann unsere Studie darstellen. Ofatumumab genießt bei unseren Patienten eine hohe Verträglichkeit und Akzeptanz. Im Vergleich zu anderen Immuntherapien ermöglicht die häusliche Selbstanwendung des Medikaments darüber hinaus ein großes Maß an Selbstständigkeit und Unabhängigkeit von stationären Klinikaufenthalten und langen Aufenthalten in Infusionsambulanzen. Insbesondere in Zeiten immer knapperer Ressourcen im Gesundheitssystem, mangelnder Pflege- und Bettenkapazität sowie pandemiebedingter Einschränkungen in Gesundheitseinrichtungen kann mit Ofatumumab eine frühe und adäquate Langzeitimmuntherapie gewährleistet werden. Die langfristigen Therapieeffekte sind mit dieser Studie zwar durch den noch kurzen Stichprobenzeitraum von einem Jahr nicht vorhersehbar, es zeichnen sich jedoch jetzt schon positive Effekte ab.

### Limitationen

Limitationen unserer Studie sind der noch kurze Stichprobenzeitraum von einem Jahr. Die subjektiven Fragebögen wurden nicht validiert und die Quote an strukturiert erfassten Patienten ist weiter steigerungsfähig. Darüber hinaus können die „patient-reported outcomes“ nach einer durchschnittlichen Therapiedauer von ca. 3 Monaten nicht mehr als Ausgangswert vor Therapie genutzt werden. Ein Zentrumseffekt ist in dieser Kohorte wahrscheinlich.

## Fazit für die Praxis

- Mit Ofatumumab steht den Patienten ein hochaktives MS-Medikament zur Selbstapplikation zur Verfügung. Die Anwendung von Ofatumumab fällt den Patienten in unserer Kohorte sehr leicht.
- Ofatumumab genießt bei unseren Patienten eine hohe Verträglichkeit und Akzeptanz.
- Die Nebenwirkungen von Ofatumumab treten hauptsächlich bei Erstanwendung auf und fallen überwiegend leicht aus.
